# Malignant Transformation and Progression of Musculoskeletal Lesions with Imaging–Pathology Correlation—Part 2: Soft Tissue Lesions

**DOI:** 10.3390/diagnostics16121782

**Published:** 2026-06-09

**Authors:** Hyang Sook Jeong, Seul Ki Lee, Jee-Young Kim, Changyoung Yoo, Min Wook Joo

**Affiliations:** 1 Department of Hospital Pathology, St. Vincent’s Hospital, College of Medicine, The Catholic University of Korea, Seoul 16247, Republic of Korea; 2 Department of Radiology, St. Vincent’s Hospital, College of Medicine, The Catholic University of Korea, Seoul 16247, Republic of Korea; 3 Department of Orthopaedic Surgery, St. Vincent’s Hospital, College of Medicine, The Catholic University of Korea, Seoul 16247, Republic of Korea

**Keywords:** malignant transformation, soft tissue lesions, imaging findings, pathology, magnetic resonance imaging

## Abstract

**Background/Objectives:** Malignant transformation of soft tissue lesions is uncommon but represents a significant diagnostic challenge with substantial clinical consequences. This spectrum encompasses four interrelated processes but biologically distinct processes: (1) true malignant transformation of benign lesions; (2) dedifferentiation of low-grade or intermediate malignancies; (3) secondary malignancy arising in chronic inflammatory or non-neoplastic conditions; and (4) apparent progression related to tumor heterogeneity and sampling error. Although these four entities involve biologically distinct mechanisms, they are grouped under “malignant progression” for conceptual clarity. While this umbrella approach has limitations due to biological heterogeneity, this unified radiologic framework aims to supplement, rather than oversimplify, their distinct biological behaviors. Representative examples include neurofibroma and epidermal inclusion cyst among benign lesions; atypical lipomatous tumor/well-differentiated liposarcoma, dermatofibrosarcoma protuberans, and solitary fibrous tumor among lesions showing dedifferentiation or malignant progression; and chronic inflammatory or scar-related conditions and previously irradiated tissue associated with secondary malignancy. Some lesions that appear to progress during follow-up may represent initial underdiagnosis rather than true biologic progression. **Methods:** This narrative review summarizes current imaging features, underlying pathologic mechanisms, and clinical risk factors associated with these processes in soft tissue lesions. Particular emphasis is placed on radiologic–pathologic correlation and conditions prone to histopathologic misinterpretation. **Results:** Imaging red flags—including interval or rapid growth, deep fascial invasion, heterogeneous enhancement, perilesional edema, and necrosis—should raise concern for malignant progression across these categories. However, overlapping imaging features and sampling errors may result in pathologic misdiagnosis and delayed treatment. Particularly, atypical lipomatous tumors are frequently misdiagnosed as simple lipomas, while fibrosarcomas may be erroneously interpreted as aggressive fibromatosis. Advanced imaging and multidisciplinary review may help reduce diagnostic errors. Patients with predisposing factors such as genetic syndromes, chronic inflammation, prior burns, or previous radiation exposure warrant close surveillance. **Conclusions:** Accurate diagnosis of soft tissue lesions with true malignant transformation, dedifferentiation, or secondary malignancy—as well as recognition of diagnostic pitfalls—is essential for appropriate management. Integrated radiologic–pathologic assessment may help improve diagnostic accuracy and clinical decision-making in soft tissue oncology.

## 1. Introduction

Common soft tissue lesions are frequently benign or indolent; however, a subset may undergo true malignant transformation, dedifferentiation, or secondary malignant change, creating significant diagnostic challenges in clinical practice. Although many benign and low-grade soft tissue tumors have characteristic imaging findings, these biologic changes may manifest with subtle or evolving imaging findings that are easily overlooked during surveillance or initial evaluation.

Histopathologic confirmation is essential but may be limited by sampling error, tumor heterogeneity, or misinterpretation, especially when malignant components are focal. As a result, biopsy findings may falsely suggest benign disease, underscoring the importance of integrated imaging–pathology correlation.

The goal of this review article is to highlight the critical importance of recognizing true malignant transformation, dedifferentiation, and secondary malignancy in soft tissue lesions and emphasize that systematic integration of imaging and pathology can enhance diagnostic accuracy and improve clinical outcomes in soft tissue oncology.

The entities discussed in this review are organized into four categories: benign tumorous lesions with (1) true malignant transformation, (2) low-grade or intermediate malignancies with dedifferentiation, (3) chronic inflammatory or treatment-related non-neoplastic conditions associated with secondary malignancy, and (4) diagnostic pitfalls caused by tumor heterogeneity and sampling error. Although these entities are discussed together under the broad educational framework of “malignant progression,” they represent biologically distinct mechanisms. Therefore, this terminology should not be interpreted as implying a single unified biologic pathway.

## 2. Methodology

This article is a narrative review synthesizing current evidence on true malignant transformation, dedifferentiation, secondary malignancy, and diagnostic pitfalls in soft tissue lesions, rather than as a formal systematic review or meta-analysis. A comprehensive literature search was performed using PubMed, Scopus, and Google Scholar databases for articles published in English between 2000 and 2025, using key terms including “malignant transformation”, “dedifferentiation”, “secondary malignancy”, and “soft tissue tumor”. Articles were selected based on clinical relevance, imaging–histologic concordance, and educational value. Case reports, review articles, and representative original studies relevant to soft tissue oncology were included. Non-English articles and studies lacking sufficient imaging or pathologic correlation were excluded.

Institutional clinical cases were retrospectively identified from the PACS archive from St. Vincent’s Hospital between 2009 and 2025. These cases were selected based on (1) histopathologically confirmed diagnosis and (2) availability of complete radiologic–pathologic correlation, enabling educational illustration. Representative cases were preferentially selected to illustrate the major conceptual categories and diagnostic pitfalls discussed in this review. Cases were initially identified through diagnosis-based PACS searches using pathologically confirmed malignant soft tissue tumor entities relevant to this review topic. Cases lacking histologic slides, adequate pathologic material, or confirmatory studies were excluded. Final inclusion was based on the availability of adequate imaging and histologic correlation and educational value for demonstrating the discussed concepts and potential diagnostic pitfalls.

All imaging data were fully anonymized prior to analysis and publication. Only patient age and sex were retained, as these are considered clinically relevant contextual variables. No identifiable patient information such as facial features, names, dates of birth, medical record numbers, or any other identifiable personal information was included in the manuscript figures or text.

Institutional review board approval was obtained solely for the use of de-identified retrospective clinical images (Approval No.: VC25RISI0302). The requirement for the informed consent, including consent for publication, was waived by the IRB in accordance with institutional policy because the study used fully anonymized retrospective data and posed minimal risk to participants.

No generative AI tools were used for data analysis, image generation, or scientific interpretation. AI-assisted language editing tools were used only for minor English grammar and language refinement, and all scientific content was reviewed and verified by the authors.

This review represents Part 2 of a two-part series addressing malignant progression in musculoskeletal lesions. While Part 1 focused on bone lesions [1], the present article concentrates on soft tissue lesions.

In this review, the term “malignant progression” is used as an umbrella concept encompassing: (1) true malignant transformation of benign lesions, (2) dedifferentiation of low-grade malignancies, (3) secondary malignancy arising in chronically inflamed non-neoplastic conditions, and (4) apparent progression related to diagnostic pitfalls caused by spatially variable tumor composition and sampling error. This terminology is used primarily for educational and practical diagnostic purposes rather than to imply a uniform biologic mechanism across all entities discussed.

## 3. Benign Soft Tissue Lesions with Malignant Potential: Tumorous Conditions

### 3.1. Benign Peripheral Nerve Sheath Tumor: Neurofibroma and Schwannoma

Neurofibroma and schwannoma are benign peripheral nerve sheath tumors (PNSTs) that typically exhibit characteristic imaging features reflecting their neural origin [2]. Although malignant peripheral nerve sheath tumor (MPNST) most commonly arises from plexiform neurofibroma in patients with neurofibromatosis type 1 (NF1), rare cases associated with schwannoma have also been reported [3].

**Figure 1:**
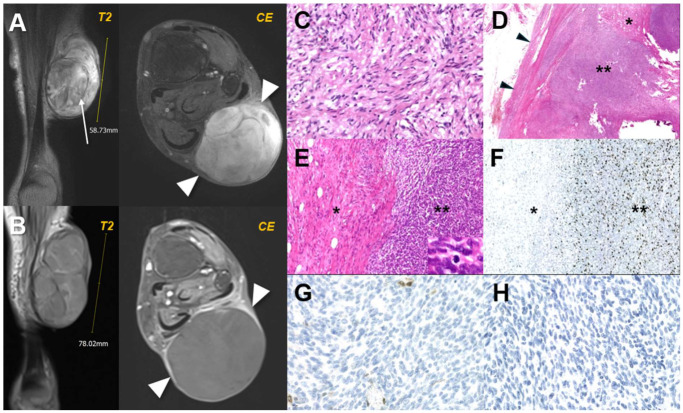
Malignant transformation of a neurofibroma in an elderly patient with neurofibromatosis type 1. (**A**) Initial left ankle MRI (9 months earlier) of coronal T2-weighted image and enhanced T1-weighted fat-suppressed image show about 5.8 cm sized multinodular soft tissue mass with a fascicular sign (arrow) and relatively homogeneous enhancement (arrowheads) in the posterolateral ankle. (**B**) Follow-up ankle MRI of coronal T2-weighted image and enhanced T1-weighted fat-suppressed image shows interval growth of the mass to 7.8 cm, with newly developed intratumoral necrotic change showing non-enhancement (arrowheads). (**C**) Initial microscopic image shows interlacing spindle cells with wavy nuclei in a wire-like collagenous stroma, consistent with a neurofibroma (H&E, ×200). (**D**) Follow-up low-power photomicrograph (8 months) of the enlarged left ankle mass demonstrates an infiltrative margin (arrowhead) with alternating hypocellular, collagenous areas (asterisk) and densely cellular spindle cell areas (double asterisks) (H&E, ×10). (**E**,**F**) High-power views show transition from conventional neurofibroma (asterisk) composed of bland spindle cells in a collagenous stroma with a low Ki-67 labeling index (<1%), to a hypercellular pleomorphic spindle cell component with fascicular growth with an increased mitotic count (10/10 HPFs; inset ×400) and an elevated Ki-67 labeling index (~30%) (double asterisks) (H&E and Ki-67 immunohistochemistry, ×100). (**G**,**H**) Decreased p16 immunoreactivity and loss of H3K27me3 expression are observed (p16 and H3K27me3 immunohistochemistry, ×400). These findings support malignant transformation to malignant peripheral nerve sheath tumor.

MPNST represents a critical clinical event, particularly in patients with NF1 (Figure 1), and a major cause of disease-related mortality [4]. Individuals with NF1 have an approximately 8–13% lifetime risk of developing MPNST [5], whereas MPNST remains extremely rare in non-NF1 populations. This process reflects a multistep molecular evolution, initiated by *NF1* loss and RAS pathway activation, with subsequent *CDKN2A/CDKN2B* inactivation and PRC2 dysfunction, most commonly involving *SUZ12* or *EED*, leading to loss of H3K27 trimethylation [6]. Clinically, new pain, rapid enlargement, or neurologic deficits should raise suspicion for malignant transformation and prompt imaging evaluation [7].

Malignant transformation of a neurofibroma on imaging is suggested by loss of target sign, marked internal heterogeneity, and ill-defined margins, often accompanied by intratumoral necrosis or hemorrhage manifested as non-enhancing areas (Figure 1) [4]. Advanced imaging techniques may increase diagnostic confidence, as restricted diffusion on diffusion-weighted imaging (DWI) reflects increased cellularity [8]. Quantitative imaging parameters may further improve diagnostic accuracy. In particular, lower ADC value (commonly < 1.0 × 10^−3^ mm^2^/s) and increased FDG uptake on PET/CT (SUVmax > 3.5–4.0) have been associated with MPNST and may help differentiate malignant transformation from benign PNSTs [9].

Histopathologically, malignant transformation occurs along a continuum rather than as an abrupt event. This process is characterized by increasing cellularity, nuclear enlargement, hyperchromasia, architectural disorganization, and rising mitotic activity. Lesions may evolve from atypical neurofibromatous neoplasms or low-grade lesions to fully developed MPNSTs characterized by diffuse hypercellularity, fascicular spindle cell architecture, geographic necrosis, and brisk mitoses (Figure 1) [10].

### 3.2. Epidermal Inclusion Cyst

Epidermal inclusion cyst (EIC) is a common benign cutaneous or subcutaneous lesion formed by proliferation of keratinizing epidermal epithelium with accumulation of laminated keratinous debris. It typically presents as a well-circumscribed, round or ovoid dermal or subcutaneous mass, showing low to intermediate echogenicity with posterior acoustic enhancement and internal lamellated echoes on ultrasound [11], and low T1-weighted and high T2-weighted signal on MRI with diffusion restriction on DWI; contrast enhancement is absent or limited to a peripheral rim [12,13].

Malignant transformation, although rare, most commonly results in squamous cell carcinoma and is thought to be driven by chronic inflammation, repeated rupture, or long-standing irritation leading to cumulative DNA damage [14]. This process has been associated with p53 pathway dysregulation and increased proliferative activity [15]; however, the precise molecular mechanisms remain incompletely understood due to its rarity.

**Figure 2:**
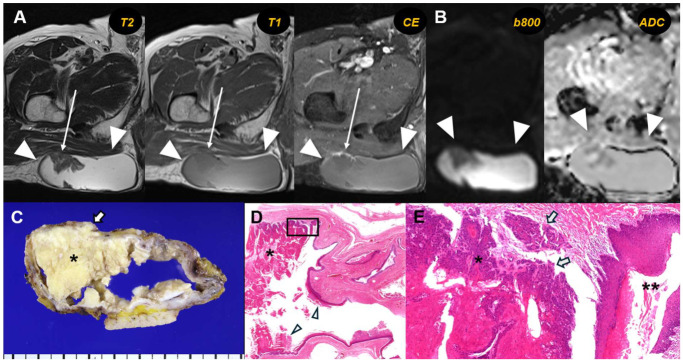
Squamous cell carcinoma arising from an epidermal inclusion cyst (EIC) in an elderly patient. (**A**) Pelvic bone MRI of axial T2- and T1-weighted image and enhanced T1-weighted fat-suppressed image demonstrate about 10 cm sized subcutaneous cystic mass (arrowheads) with homogeneous signal intensity and a focally enhancing mural solid component (arrow). (**B**) Axial DWI of *b* value 800 and ADC map shows diffusion restriction in the cystic content (arrowheads). (**C**) Gross photograph of the subcutaneous lesion shows a unilocular cyst with a thick fibrotic wall and an intraluminal exophytic mass (asterisk) with a focal area of perilesional soft tissue invasion (arrow). (**D**) Low-power photomicrograph shows an EIC lined by benign squamous epithelium (upper right and bottom) with laminated keratin (arrowhead). An associated squamous cell carcinoma (asterisk) is present as a markedly keratinized, exophytic papillary mass contiguous with the benign cyst wall (boxed area; see panel **D**) (H&E, ×10). (**E**) Transition area from EIC (double asterisks) to squamous cell carcinoma (asterisk), with nuclear atypia and irregular tumor nests infiltrating the surrounding soft tissue (arrow) (H&E, ×200).

Imaging features concerning malignant transformation include rapid interval growth, increased internal heterogeneity, and irregular or nodular wall thickening with enhanced solid components, often accompanied by invasion of adjacent soft tissues (Figure 2) [16]. Although cyst rupture may produce wall thickening, edema, and inflammatory enhancement, these reactive changes typically lack discrete solid enhancing components [17]. Careful assessment of lesion margins and enhancing patterns is essential to distinguish malignant change from benign inflammatory processes [18]. Inflamed or ruptured EIC may show wall thickening and peripheral enhancement, mimicking malignancy; therefore, clinicopathologic and imaging correlation remains important [12]. Rapid growth or enhancing mural nodules should raise suspicion for squamous cell carcinoma arising in EIC.

Pathologically, malignant transformation is characterized by invasive proliferation of atypical squamous cells arising from the cyst wall, showing nuclear pleomorphism, prominent nucleoli, increased mitoses, and areas of necrosis [19]. Identification of a transition zone between benign cyst epithelium and malignant squamous components supports the diagnosis of carcinoma arising in EIC (Figure 2).

Additional benign soft tissue tumors with rare reported malignant transformation include tenosynovial giant cell tumor and glomus tumor [20,21]. Although uncommon, malignant variants of these entities have been described and may demonstrate locally aggressive behavior, recurrence, or metastatic potential. Because of their rarity and overlapping histologic features, diagnosis often requires careful radiologic–pathologic correlation and exclusion of other high-grade sarcomas.

## 4. Low-Grade or Intermediate Malignancies with Dedifferentiation

### 4.1. Atypical Lipomatous Tumor/Well-Differentiated Liposarcoma with Dedifferentiation

Atypical lipomatous tumor/well-differentiated liposarcoma (ALT/WDL) typically presents as a slow-growing, deep-seated lipomatous mass composed predominantly of mature fat. On imaging, ALT/WDL characteristically demonstrates thickened or nodular septa (often >2 mm), nodular non-adipose components, and mild-to-moderate septal enhancement, while overall preserving a fatty architecture [22].

Dedifferentiation is suspected when ALT/WDL develops a newly enlarging, solid non-lipomatous component that is disproportionate to the fatty portion or shows rapid interval growth [23,24]. Clinically increasing pain, accelerated enlargement, or recurrence after marginal resection should prompt concern for transformation toward dedifferentiated liposarcoma [25,26]. The natural history and prognosis of extremity ALT remain incompletely understood, and the rate of progression to dedifferentiated liposarcoma appears to be substantially lower than that observed in the retroperitoneal WDLS [24]. Dedifferentiation is rare in extremity ALT (<5%) but more common in retroperitoneal WDLS (~15–20%, up to 30% with long-term follow-up), particularly after repeated local recurrence [27,28]. Retroperitoneal tumors are often larger at presentation and more difficult to completely resect, resulting in frequent local recurrence and increased risk of dedifferentiation. Dedifferentiated liposarcoma retains *MDM2* and *CDK4* amplification of the 12q13-15 locus. Additional genomic instability contributes to loss of adipocytic differentiation and development of aggressive sarcomatous features [29].

**Figure 3:**
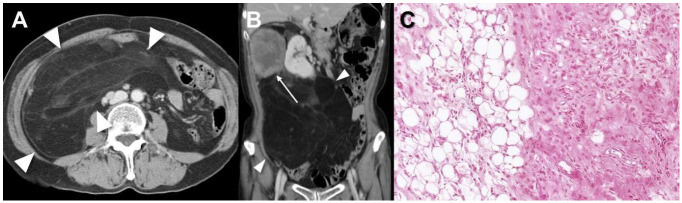
Dedifferentiated liposarcoma in an adult patient. (**A**) Axial contrast-enhanced CT image shows a large, predominantly low-attenuation mass (arrowheads), consistent with a lipomatous tumor in the retroperitoneum. (**B**) Coronal reformatted CT image delineates a discrete, enhancing non-lipomatous solid component (arrow) within a well-differentiated liposarcoma (arrowheads). (**C**) Microscopic image of the retroperitoneal mass shows an atypical lipomatous tumor component (left), with pleomorphic adipocytes and atypical stromal cells within fibrous septa, abruptly transitioning to sheets of pleomorphic spindle cells resembling high-grade sarcoma (right), consistent with dedifferentiated liposarcoma (H&E, ×400).

Dedifferentiated liposarcoma typically appears on imaging as a predominantly fatty mass consistent with ALT/WDL, containing a newly developed, discrete non-lipomatous solid component [30,31]. On MRI, the dedifferentiated portion demonstrates low signal on T1-weighted image, intermediate to high signal on T2-weighted image, and heterogeneous or avid enhancement, sharply contrasting with the surrounding fatty component [30,31,32]. CT often reveals a focal soft tissue nodule with attenuation similar to muscle within a lipomatous background, sometimes producing a characteristic “mosaic” appearance (Figure 3) [30,33].

Pathologically, dedifferentiated liposarcoma is characterized by the presence of a non-lipogenic high-grade sarcomatous component arising adjacent to or within a WDLS. The dedifferentiated areas typically show increased cellularity, nuclear atypia, and mitotic activity, often resembling undifferentiated pleomorphic sarcoma or high-grade spindle cell sarcoma. An abrupt transition from the well-differentiated adipocytic component to the dedifferentiated sarcomatous component is a characteristic diagnostic finding (Figure 3) [24,32,34].

### 4.2. Dermatofibrosarcoma Protuberans with Fibrosarcomatous Transformation

Dermatofibrosarcoma protuberans (DFSP) is a rare, low-to-intermediate-grade dermal/subcutaneous fibroblastic neoplasm characterized by locally aggressive, infiltrative extension into the subcutaneous tissue and a high propensity for local recurrence [35]. On imaging, DFSP commonly exhibits a tail-like or lace-like pattern along the subcutaneous septa, reflecting infiltrative growth [36]. Although these imaging features are not specific, the combination of superficial location, infiltrative margins, and avid contrast enhancement is suggestive of the diagnosis [35].

Dedifferentiation of DFSP most often occurs as fibrosarcomatous transformation, which is clinically associated with more aggressive behavior, including increased rates of local recurrence and a higher risk of metastasis. This progression may be suspected in cases showing rapid tumor growth or repeated local recurrence [37]. Both conventional and fibrosarcomatous DFSP typically harbor the *COL1A1-PDGFB* fusion, indicating a shared molecular origin despite divergent clinical behavior [38].

**Figure 4:**
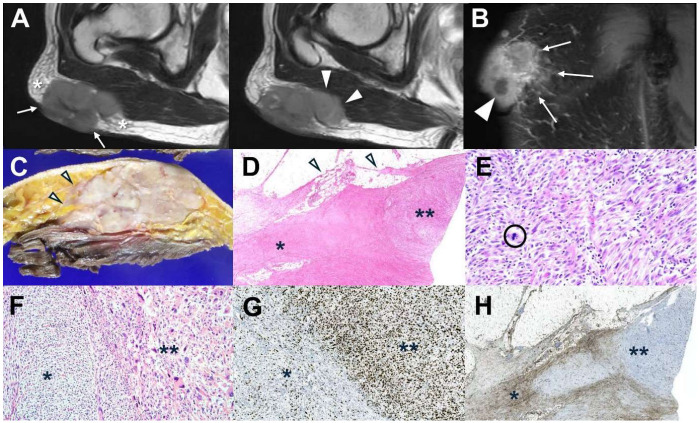
Fibrosarcomatous transformation of dermatofibrosarcoma protuberans (DFSP) in an elderly patient. (**A**) Two consecutive axial T2-weighted images demonstrate a superficial soft tissue mass arising from the skin (arrows), infiltrating the subcutaneous fat (asterisks), with focal extension into the adjacent muscle (arrowheads). (**B**) Coronal contrast-enhanced fat-suppressed T1-weighted image shows the mass with infiltrative margin (arrows) and heterogeneous enhancement with internal non-enhancing portion (arrowhead), suggesting necrotic change. (**C**) Gross photograph shows an expansile mass involving dermis and subcutaneous fat, with a firm, gray-white, lobulated cut surface and radiating fibrous bands (arrowhead). (**D**) Low-power view reveals a relatively well circumscribed, densely cellular component (double asterisks) within the DFSP lesion (asterisk), showing infiltrating tentacle-like growth into the surrounding subcutaneous tissue, with a honeycomb appearance (arrowhead) (H&E, ×15). (**E**) High-grade spindle cell sarcoma with herringbone pattern and increased mitotic activity (7/10 HPFs), including atypical forms (circled), consistent with fibrosarcomatous transformation (H&E, ×400). (**F**,**G**) Transitional area from DFSP (asterisk) to high-grade sarcoma (double asterisks) with increased nuclear atypia and Ki-67 labeling index (10% → 93%) (H&E and Ki-67, ×200). (**H**) Attenuated CD34 expression in the high-grade component (double asterisks) supports fibrosarcomatous transformation of DFSP, within the DFSP lesion (asterisk) (same field as **D**) (CD34, ×15).

From a radiologic perspective, fibrosarcomatous dedifferentiation of DFSP is suggested by imaging features indicative of increased aggressiveness. Compared with conventional DFSP, transformed lesions tend to show deeper infiltration, frequently into the deep fascia or adjacent muscle (Figure 4) [39]. On MRI, these tumors commonly show markedly internal heterogeneity, often with necrotic, hemorrhagic, or cystic components (Figure 4) [40].

Pathologically, fibrosarcomatous dedifferentiation of DFSP most commonly occurs as fibrosarcomatous change, marked by an abrupt or gradual transition from conventional storiform DFSP showing infiltration of the subcutaneous fat in a honeycomb pattern to a high-grade component composed of densely cellular, pleomorphic spindle cells arranged in a herringbone pattern, with increased nuclear atypia and mitotic activity (Figure 4) [41].

### 4.3. Solitary Fibrous Tumor with Dedifferentiation

**Figure 5:**
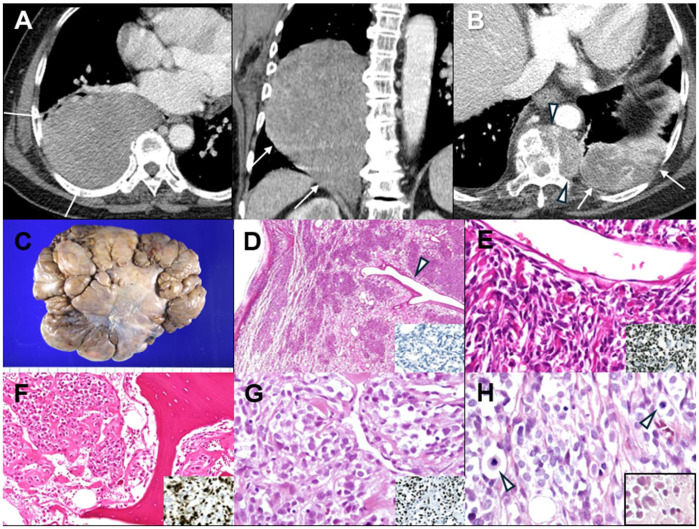
Malignant transformation of solitary fibrous tumor (SFT) with lung and bone metastasis. (**A**) Contrast-enhanced axial and coronal CT images in an elderly patient demonstrates a well-circumscribed, heterogeneously enhancing pleural-based mass (arrows), proven as SFT by biopsy. (**B**) Five years later, follow-up axial contrast-enhanced CT image shows a heterogeneously enhancing mass representing pulmonary metastasis (arrows) with direct invasion into the adjacent vertebral body (arrowheads), compatible with malignant SFT and metastasis. (**C**) Initial gross photograph of the surgically excised, exophytic tumor (17.0 cm) with glistening surface covered by pleura. (**D**) Low-power view reveals alternating hypercellular and hypocellular areas with prominent hyalinized staghorn vessels (arrowhead) and a low Ki-67 labeling index (<1%) (inset, ×200) (H&E, ×20). (**E**) Patternless spindle-cell proliferation with diffuse nuclear STAT6 expression supports the diagnosis of SFT (inset, ×200) (H&E, ×400). (**F**) Follow-up spinal lesion identified 5 years later shows malignant SFT, composed of tumor cells with bone invasion and an increased Ki-67 index (35%) (inset, ×200) (H&E, ×100). (**G**) High-power view shows haphazardly arranged spindle to ovoid cells with branching hyalinized vessels and diffuse nuclear STAT6 positivity, confirming SFT (inset, ×200) (H&E, ×400). (**H**) Increased mitotic activity (arrowhead; 5/10 HPFs) and tumor necrosis (inset, ×400) support the diagnosis of malignant SFT (H&E, ×400).

Solitary fibrous tumor (SFT) is a rare mesenchymal neoplasm of fibroblastic origin that can arise commonly from the pleura but also in a wide range of extrapleural soft tissues (Figure 5) [42]. It is typically a slow-growing and well-circumscribed mass, but exhibits a spectrum of biologic behavior ranging from indolent lesions to intermediate tumors with malignant potential. On imaging, SFT usually appears as a well-defined, lobulated solid mass with intense enhancement, reflecting hypervascularity and prominent feeding vessels [43]. The tumor contains marked T2 hypointensity corresponding to dense collagenous stroma on MRI. Flow voids or serpentine vessels may be presented [44].

Sudden enlargement or new-onset pain in a previously indolent SFT should raise suspicion for dedifferentiation. Malignant SFTs also carry a higher risk of local recurrence and distant metastasis, most commonly to the lung, liver, and bone (Figure 5) [43]. The molecular hallmark of SFT is the recurrent *NAB2-STAT6* fusion, generated by an intrachromosomal inversion on chromosome 12q13, which promotes tumorigenesis through dysregulated transcriptional activity [45]. Malignant progression may involve additional alterations such as *TP53* mutations and *TERT* promoter mutations [46].

Dedifferentiation should be suspected when a previously well-circumscribed SFT develops aggressive imaging features. These include increased heterogeneity, necrosis, hemorrhage, or infiltrative margins. MRI may demonstrate increased heterogeneity with mixed T2 signal intensity and irregular enhancement. Imaging evidence of adjacent organ invasion, bone destruction, or distant metastasis should further raise concern for malignant behavior (Figure 5) [44,47]. Histologic findings may underestimate biologic aggressiveness in some SFTs. Therefore, longitudinal imaging follow-up and multidisciplinary assessment remain important after diagnosis. Malignant SFT demonstrates increased cellularity, pleomorphism, infiltrative growth, and necrosis and elevated mitotic activity [48]. These findings contrast with the collagen-rich patternless architecture of conventional SFT (Figure 5) [48].

## 5. Non-Neoplastic Lesions with Secondary Malignancy

### 5.1. Chronic Empyema-Associated Angiosarcoma

**Figure 6:**
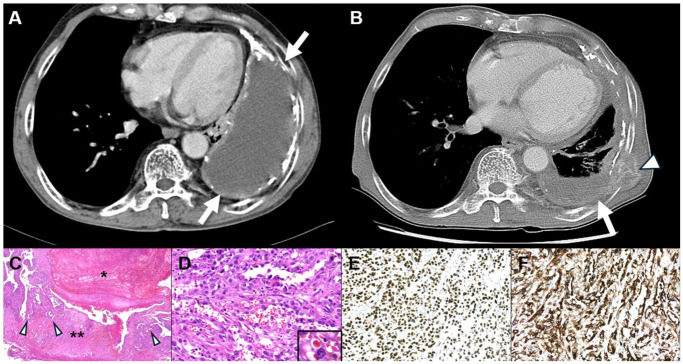
Angiosarcoma arising in long-standing chronic empyema. (**A**) Initial contrast-enhanced chest CT demonstrates a chronic empyema cavity in the left hemithorax with associated pleural thickening and fluid collection (arrows) with a 30-year history of TB pleurisy. (**B**) Follow-up contrast-enhanced chest CT obtained 2 years later shows interval development of an irregular, heterogeneously enhancing soft tissue mass (arrowhead) from the empyema cavity (arrow) with chest wall involvement, consistent with malignant soft tissue tumor. (**C**) The specimen was obtained from the pleural mass observed on follow-up chest CT and low-power microscopic view shows a hemorrhagic and fibrinous material (asterisk) overlying a thickened pleural wall infiltrated by tumor cells (double asterisks) forming thin vascular spaces (arrowhead) (H&E, ×20). (**D**) High-power view of the pleural wall reveals epithelioid, pleomorphic tumor cells with intracytoplasmic lumina containing red blood cells (inset, ×400), forming irregular, anastomosing vascular channels infiltrating the pleural tissue (H&E, ×400). (**E**,**F**) Diffuse ERG and CD31 expression in the tumor cells, supporting epithelioid angiosarcoma (ERG and CD31, ×200).

Chronic empyema-associated angiosarcoma is a rare but highly aggressive malignancy that develops in the setting of long-standing pleural inflammation, most often decades after tuberculous pleuritis or therapeutic pneumothorax (Figure 6) [49]. Clinically, patients typically present with nonspecific symptoms such as chest pain, dyspnea, or a rapidly enlarging chest wall mass, frequently leading to delayed diagnosis after a prolonged latency period of 20–30 years (Figure 6) [50]. Persistent inflammation, chronic hypoxia, repeated hemorrhage, and cytokine-driven endothelial proliferation are thought to create a microenvironment conducive to secondary malignant change [51]. Angiosarcoma itself is a malignant tumor of endothelial origin characterized by aggressive behavior and high metastatic potential [52]. At the molecular level, although data are limited, *MYC* amplification—particularly in secondary angiosarcoma—and genomic instability have been implicated in its pathogenesis [53].

On imaging, chronic empyema-associated angiosarcoma often presents as an aggressive, infiltrative soft tissue mass along the pleural cavity or chest wall, frequently associated with pre-existing pleural thickening, calcification, or chronic empyema cavity (Figure 6). CT may demonstrate a heterogeneously enhancing mass with areas of necrosis and hemorrhage, often accompanied by rib destruction or chest wall invasion (Figure 6) [54]. MRI typically reveals heterogeneous signal intensity with mixed high and low signal on T2-weighted image due to hemorrhage and necrosis and variable enhancement after contrast administration [55]. These findings are nonspecific but, in the appropriate clinical context of long-standing empyema, should raise suspicion for secondary angiosarcoma arising in chronic empyema [56].

Histologically, chronic empyema-associated angiosarcoma is characterized by malignant endothelial proliferation forming irregular, anastomosing vascular channels with marked cytologic atypia, high mitotic activity, and frequent hemorrhage and necrosis [57]. Poorly differentiated areas may exhibit solid growth with loss of vasoformative features (Figure 6). Immunohistochemically, tumor cells express endothelial markers including CD31, CD34, ERG, and FLI1, with CD31 being the most sensitive and specific [57]. The tumor is thought to arise in the setting of long-standing chronic inflammation and hemorrhage within the empyema cavity.

### 5.2. Pyothorax-Associated Lymphoma

Pyothorax-associated lymphoma (PAL) is a rare subtype of extranodal non-Hodgkin lymphoma that arises in the setting of long-standing chronic pyothorax, most commonly following tuberculous pleuritis or artificial pneumothorax therapy [58]. It is now classified as a subtype of diffuse large B-cell lymphoma (DLBCL) associated with chronic inflammation in the current WHO classification [59]. PAL typically develops after a prolonged latency period, often exceeding 20–40 years [60]. Chronic inflammatory stimulation and immune dysregulation are considered important contributors to lymphomagenesis. At the molecular level, Epstein–Barr virus (EBV) infection plays a central role, with most tumors showing EBV positivity and expression of EBV-encoded RNA (EBER), suggesting that chronic inflammation combined with local immunosuppression facilitates clonal B-cell proliferation [61]. Clinically, patients often present with chest pain, fever, or a progressively enlarging chest wall mass, and systemic “B symptoms”, may also be observed [62].

**Figure 7:**
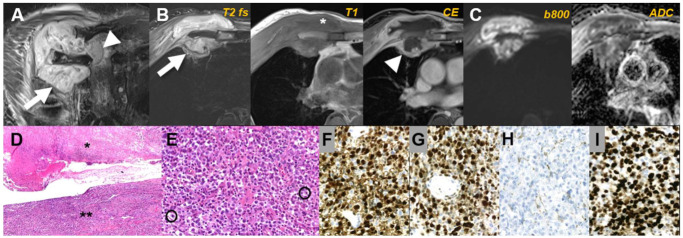
Pyothorax-associated lymphoma involving the chest wall. (**A**) Coronal T2-weighted fat-suppressed image shows a heterogeneously hyperintense soft tissue mass at the chest wall with invasion into the sternum (arrowhead). (**B**) Axial T2 fat-suppressed image shows a heterogeneously hyperintense, lenticular-shaped soft tissue mass along the chest wall and pleura (arrow). T1-weighted image reveals an iso-to-hypointense mass with internal heterogeneous low signal intensity areas (asterisk), suggesting necrosis. Contrast-enhanced (CE) image shows a necrotic change with enhancing solid portion (arrowhead). (**C**) Diffusion-weighted imaging with b800 demonstrates high signal intensity of the lesion. Corresponding ADC map shows low signal intensity, consistent with diffusion restriction. (**D**) Low-power view shows inflammatory exudates with fibrinous material (asterisk) and an underlying fibrous pleural wall with a diffuse infiltrate of lymphoid cells (double asterisks) (H&E, ×10). (**E**) High-power photomicrograph of the pleural wall shows sheets of large atypical lymphoid cells with prominent nucleoli and increased mitotic activity (circled; 14/10 HPFs) (H&E, ×200). (**F**) Immunohistochemistry reveals diffuse CD20 expression in the tumor cells, confirming a malignant lymphoma of B-cell lineage. (**G**) MUM1 positivity and (**H**) absence of CD10 expression support a non-GCB phenotype (CD20, MUM1, and CD10, ×400). (**I**) Ki-67 demonstrates a high proliferative index (~73%) (×400). EBER in situ hybridization was negative. Findings support diffuse large B-cell lymphoma with chronic inflammation (DLBCL-CI) (EBV-negative).

On imaging, PAL typically manifests as a soft tissue mass arising along the pleural cavity, often adjacent to a chronic empyema space with associated pleural thickening and calcification. CT usually demonstrates a lobulated or infiltrative mass with relatively homogeneous or mildly heterogeneous enhancement, frequently accompanied by chest wall invasion or rib destruction (Figure 7) [62]. MRI generally shows intermediate to high signal intensity on T2-weighted image and iso-to-hypointense signal on T1-weighted image, with moderate enhancement [63]. Compared with empyema, PAL more commonly demonstrates a solid enhancing soft tissue component and aggressive features [64]. Central necrosis or cavitary change may also be present, occasionally mimicking chronic empyema or other necrotic malignancies (Figure 7). In such cases, recognition of the background of long-standing pyothorax, pleural calcification, and associated soft tissue components is critical for accurate diagnosis [65]. PAL may mimic chronic empyema, organizing hematoma, or necrotic sarcoma on imaging. Awareness of this entity and integration of clinical history may facilitate timely biopsy and avoid delayed diagnosis [62].

Histologically, PAL is characterized by diffuse proliferation of large atypical lymphoid cells with vesicular nuclei, prominent nucleoli, and frequent mitotic figures, consistent with DLBCL (Figure 7) [66]. Extensive necrosis is common and reflects aggressive tumor biology. Immunohistochemically, tumor cells express B-cell markers including CD20, CD79a, and PAX5. Expression of activation markers such as *MUM1* is common, while germinal center markers are variably expressed [67]. In situ hybridization for EBV-encoded RNA is typically positive reflecting its pathogenesis associated with Epstein–Barr virus (EBV) infection [67]. EBV-negative cases arising in the setting of chronic pyothorax have been rarely reported, and such cases suggest that the chronically inflamed microenvironment itself may contribute to lymphomagenesis, even in the absence of detectable EBV [67].

### 5.3. Burn Scar-Associated Squamous Cell Carcinoma (Marjolin’s Ulcer)

**Figure 8:**
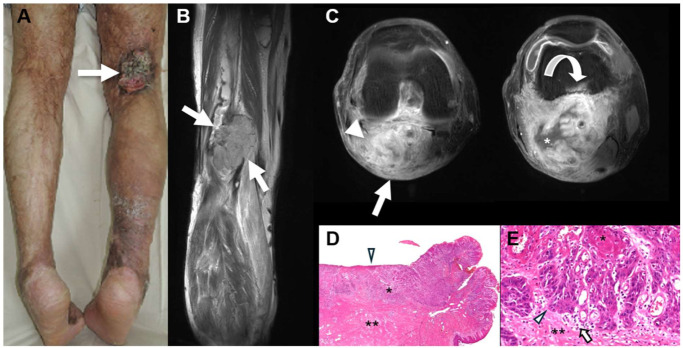
Marjolin’s ulcer, arising at a prior skin graft site following a burn injury sustained approximately 40 years earlier. (**A**) An exophytic, ulcerated mass (arrow) arising in a burn scar of the left popliteal area, with everted margins and associated post-burn contracture of the surrounding skin. (**B**) Coronal T2-weighted image demonstrates an ill-defined, heterogeneously high signal intensity mass (arrows) involving the popliteal fossa. (**C**) Axial fat-suppressed contrast-enhanced T1-weighted image shows a heterogeneously enhancing infiltrative soft tissue mass with focal central non-enhancement, suggestive of necrosis (asterisk), extending from the skin (arrow) to adjacent muscles (arrowhead) and bone (curved arrow). (**D**) Low-power view shows infiltrating tumor cells (asterisk) extending into the scar-altered dermis (double asterisks) beneath a central hemorrhagic ulcer (arrowhead) (H&E, ×10). (**E**) High-power view demonstrates moderately differentiated squamous cell carcinoma with keratinization (asterisk), forming irregular tongues (arrowhead) infiltrating fibrotic dermis (double asterisks), with adjacent chronic inflammatory infiltration (arrow) (H&E, ×400).

Burn scar-associated squamous cell carcinoma (SCC), also known as Marjolin’s ulcer, is a rare but aggressive malignancy that arises in chronically scarred or inflamed skin, most commonly following burn injury [68]. It typically develops after a prolonged latency period of several decades, often exceeding 20–30 years, reflecting the role of persistent tissue injury and inflammatory stimulation and impaired immune surveillance in carcinogenesis (Figure 8) [68]. At the molecular level, cumulative DNA damage and genomic instability induced by chronic inflammation are thought to play key roles, with reported alterations including *TP53* mutations and dysregulation of pathways involved in cell proliferation and apoptosis [69]. Reduced vascularity and lymphatic drainage within scar tissue may further contribute to a locally immunosuppressed microenvironment, facilitating malignant transformation [69]. Clinically, patients usually present with a non-healing ulcer, exophytic mass, or progressive lesion within a long-standing scar, often associated with pain, bleeding, or secondary infection [70].

On imaging, Marjolin’s ulcer typically appears as an irregular soft tissue mass or ulcerative lesion arising from the skin and subcutaneous tissues at the site of a chronic scar. CT may demonstrate a heterogeneously enhancing mass with ill-defined margins, frequently associated with skin thickening, ulceration, and invasion into underlying soft tissues, including muscle or bone in advanced cases [71]. Compared with benign chronic wounds or scar tissue, Marjolin’s ulcer more often shows a solid enhancing component, aggressive infiltration, and destruction of adjacent structures (Figure 8). MRI typically shows intermediate to high signal intensity on T2-weighted image and iso-to-low signal intensity on T1-weighted image, with heterogeneous enhancement [71]. Recognition of interval growth, new soft tissue nodularity, or increasing enhancement within a chronic scar is critical, as these findings should raise suspicion for secondary squamous cell carcinoma arising in chronic scar tissue [72]. Accordingly, a low threshold for biopsy is warranted in any chronic scar or non-healing wound demonstrating such changes. Delayed diagnosis is common because early malignant change may mimic chronic inflammatory or infectious ulceration both clinically and radiologically. Therefore, interval progression and development of enhancing soft tissue nodularity should not be dismissed as benign scar-related change [70].

Histologically, Marjolin’s ulcer is most commonly a well- to moderately differentiated SCC characterized by invasive nests and cords of atypical squamous cells with keratinization and formation of keratin pearls (Figure 8) [73]. Marked cytologic atypia, increased mitotic activity, and stromal desmoplasia are frequently observed. Poorly differentiated tumors may show reduced keratinization and more infiltrative growth pattern. Immunohistologically, tumor cells express squamous markers such as p40, p63, and cytokeratins (e.g., CK5/6) [74].

### 5.4. Radiation-Induced Soft Tissue Sarcoma

**Figure 9:**
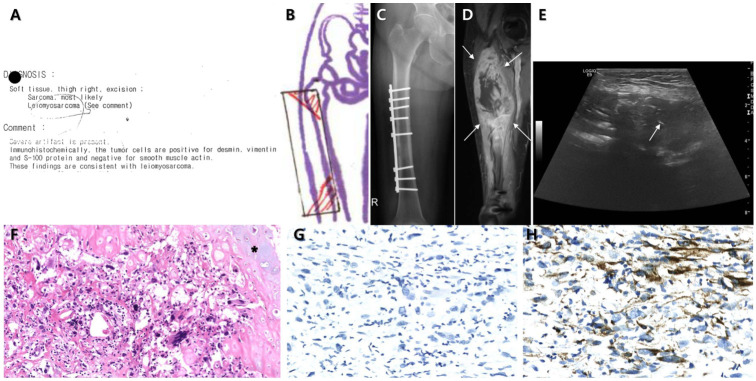
Radiation-induced sarcoma (RIS) of the right thigh. (**A**) Initial excision of a right thigh mass was performed 17 years before the diagnosis of RIS. Original pathology slides were unavailable for review; the report described the lesion as leiomyosarcoma based on immunohistochemical findings. (**B**) Schematic illustration of the postoperative external beam radiotherapy field administered to the right thigh (total dose: 65 Gy in 180 cGy fractions). (**C**) Radiograph obtained 7 years after radiotherapy demonstrating radiation-associated femoral insufficiency/pathologic fracture within the irradiated field, treated with plate-and-screw fixation. (**D**) MRI obtained 17 years after radiotherapy showing a newly developed necrotic soft tissue mass (arrows) arising within the prior radiation field. (**E**) Ultrasound-guided core needle biopsy performed for the newly developed mass. The biopsy needle tip is indicated by the arrow. (**F**) Needle biopsy of the thigh mass shows highly pleomorphic epithelioid to spindle cells with nuclear hyperchromasia, infiltrating and destroying the femoral cortex (asterisk) (H&E, ×200). (**G**) Negative desmin (Desmin, ×400). (**H**) Focal vimentin positivity without specific lineage differentiation, consistent with undifferentiated pleomorphic sarcoma (Vimentin, ×400). Given the development of a high-grade sarcoma within the prior radiation field after a 17-year latency period, the lesion was diagnosed as RIS.

Radiation-induced soft tissue sarcoma (RIS) is a rare but aggressive secondary malignancy that develops within a previously irradiated field after a prolonged latency period, typically exceeding 5–10 years (Figure 9) [75]. Chronic radiation-induced DNA damage, genomic instability, and impaired tissue repair are considered key mechanisms underlying sarcomagenesis. Histologic subtypes include undifferentiated pleomorphic sarcoma, angiosarcoma, fibrosarcoma, and osteosarcoma [76].

On imaging, RIS typically manifests as a newly developed enlarging soft tissue mass arising within an area of prior radiation change (Figure 9). MRI commonly demonstrates heterogeneous T2 hyperintensity, irregular enhancement, necrosis, and infiltrative margins [77]. Distinguishing recurrent primary malignancy from RIS may be challenging because both may occur within previously treated tissues. However, a newly developed aggressive mass after a prolonged latency period should raise suspicion for secondary sarcoma.

Histologically, radiation-induced sarcomas may arise in irradiated bone or soft tissue and are typically high-grade tumors, most commonly undifferentiated pleomorphic sarcoma or osteosarcoma [77,78]. Pathologic diagnosis requires correlation with prior radiation history and exclusion of recurrent primary malignancy (Figure 9). Microscopically, RIS often demonstrates marked cellular atypia, brisk mitotic activity, infiltrative growth, and variable necrosis, although histologic appearance depends on the sarcoma subtype [78].

Because radiation-induced fibrosis and post-operative change may obscure early tumor development, delayed diagnosis is common. Therefore, interval progression, new nodular enhancement, or development of aggressive imaging features within previously irradiated tissue should not be dismissed as post-treatment change [79].

## 6. Diagnostic Pitfalls: Tumor Heterogeneity and Sampling Error

Importantly, not all lesions that appear to transform during follow-up represent true biologic transformation or dedifferentiation. Some cases reflect initial underdiagnosis due to tumor heterogeneity, limited biopsy sampling, or subtle histologic atypia. In particular, ALTs may be misinterpreted as simple lipomas [22], while fibrosarcomas can be mistaken for aggressive fibromatosis [80]. Such erroneous interpretations may misleadingly suggest a possibility of malignant transformation, despite the lesion being malignant from the initial presentation. Radiologic–pathologic discordance should therefore be regarded as a major warning sign in soft tissue oncology. When imaging demonstrates aggressive behavior disproportionate to a benign or low-grade pathologic diagnosis, the possibility of undersampling, spatial tumor heterogeneity, or missed high-grade components should be actively considered.

**Figure 10:**
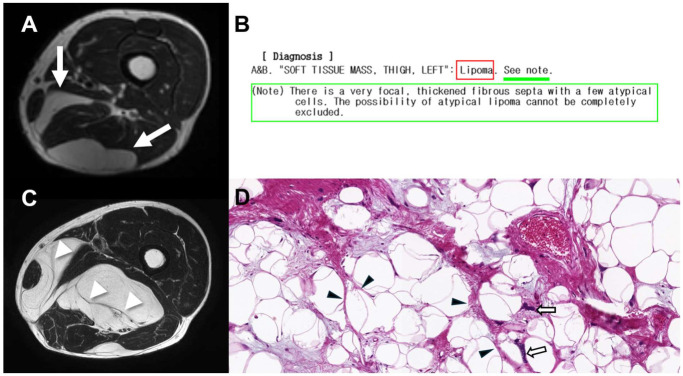
Serial imaging of an atypical lipomatous tumor, initially interpreted as lipoma 13 years earlier. (**A**) Initial axial T2-weighted image demonstrates a well-defined intermuscular fatty mass (arrows) with homogeneous signal intensity, consistent with a lipoma. (**B**) Pathologic report at that time. (**C**) Follow-up T2-weighted image shows interval re-growth with the development of thickened internal septa (arrowheads), suggestive of ALT. (**D**) High-power photomicrograph of the recurrent left thigh mass shows features of atypical lipomatous tumor/well-differentiated liposarcoma (ALT/WDL), including variably sized adipocytes and interdigitating fibrotic bands (arrowheads) with atypical stromal cells (arrow) (H&E, ×400).

In the ALT case, the initial biopsy was interpreted as consistent with a lipoma based on predominantly mature adipocytic proliferation (Figure 10). However, closer examination revealed focal nuclear atypia and the presence of fibrous septa, raising the possibility of ALT (Figure 10) [81], although the findings were insufficient for a definitive diagnosis at that time; notably, the original histologic slices were unavailable for review, and this information was based solely on medical record documentation. Following surgical excision, a recurrent lesion developed at the same site, in which lipoblasts and more conspicuous fibrous septa were identified, supporting the diagnosis of ALT (Figure 10).

Retrospectively, the initial specimen likely contained subtle atypical features, but these were limited and easily overlooked within an otherwise benign-appearing lipomatous tumor (Figure 10). This case illustrates how limited sampling and subtle histologic atypia may collectively contribute to underdiagnosis, particularly in low-grade malignancy [81]. This pitfall is particularly important in large lipomatous tumors, in which biopsy samples obtained only from fatty-appearing regions may fail to capture diagnostically significant atypical stromal cells or dedifferentiated foci. Correlation with MRI-based non-adipose components may improve diagnostic yield. Given the well-recognized risk of sampling error in large lipomatous tumors, particularly ALT/WDL with marked intratumoral heterogeneity, image-guided core needle biopsy may be insufficient for definitive diagnosis. Therefore, when clinically feasible, excisional biopsy or larger-volume tissue sampling is often recommended to allow more comprehensive histologic evaluation and reduce the risk of underdiagnosis [27].

Therefore, even subtle atypical features should raise consideration of ALT when clinical or imaging findings are concerning—including large tumor size (>10 cm), location in the lower extremity or retroperitoneum, and deep intramuscular position, or MRI features of thick fibrous septa, non-adipose nodules, or septal or nodular enhancement—in which case, *MDM2/CDK4* FISH demonstrating gene amplification supports the diagnosis [71].

**Figure 11:**
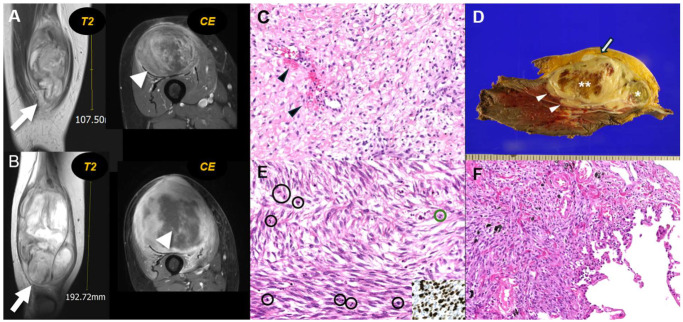
Serial imaging of a fibrosarcoma, initially misinterpreted as fibromatosis 4 months earlier. (**A**) Initial coronal T2-weighted image demonstrates a relatively well-defined soft tissue mass at the rectus femoris muscle (arrow) with intermediate to mildly high signal intensity (107.50 mm). The corresponding axial contrast-enhanced image shows heterogeneous enhancement (arrowhead). (**B**) Follow-up MRI shows marked interval enlargement of the lesion with increased heterogeneity (arrow) on coronal T2-weighted image (192.72 mm). The contrast-enhanced image shows more heterogeneous enhancement with necrotic change (arrowhead), raising suspicion for a malignant process. (**C**) Initial excisional biopsy of a right thigh mass showing fascicles of bland fibroblasts in a collagenized stroma, with thin-walled vessels and focal microhemorrhages (arrowhead), interpreted as desmoid-type fibromatosis (H&E, ×200). (**D**) Gross photograph of the recurrent thigh mass showing an expansile lesion in the rectus femoris, invading surrounding fat (arrow) and muscle (arrowhead), with a fleshy, tan cut surface and areas of necrosis (asterisk) and hemorrhage (double asterisks). (**E**) High-grade fibroblastic sarcoma with herringbone pattern, marked nuclear pleomorphism, brisk mitotic activity (black circles; 45/10 HPFs), including atypical mitoses (green circle), and elevated Ki-67 index (~64%) (inset, ×200) (H&E, ×400). (**F**) Low-power view of multiple lung nodules showing metastatic sarcoma (H&E, ×100).

In the fibrosarcoma case, the initial biopsy specimen was relatively small (approximately 0.8 cm). Although β-catenin immunohistochemistry shows no nuclear staining, rarity of high-grade fibroblastic sarcoma and the uniformly low cytologic atypia were prioritized, leading to an initial underdiagnosis as fibromatosis (Figure 11) [82].

High-grade fibrosarcomas may exhibit heterogeneous histologic features, including areas of deceptively bland spindle cell proliferation embedded in collagenous stroma, overlapping with those seen in fibromatosis (Figure 11). As a result, sampling bias toward these lower-grade-appearing regions can lead to underdiagnosis.

Desmoid-type fibromatosis is a benign but locally aggressive fibroblastic neoplasm that typically demonstrates nuclear β-catenin expression and *CTNNB1* mutation in most sporadic cases, and is commonly associated with predisposing factors such as pregnancy, prior surgery or trauma, and Familial Adenomatous Polyposis (FAP) [73]. In contrast, high-grade fibroblastic sarcomas are exceptionally rare and generally show more aggressive biologic behavior, often with greater cytologic atypia and increased proliferative activity. Notably, they may also contain relatively bland areas that can mimic fibromatosis in limited biopsy specimens, thereby creating potential for diagnostic underestimation. Although malignant transformation of desmoid-type fibromatosis into fibrosarcoma has been reported, it is exceedingly rare, with fewer than 20 cases described in the literature. Reported cases commonly involved prolonged recurrence and multiple resections; however, many predated modern immunohistochemical and molecular diagnostic techniques, making it difficult to exclude an initially underdiagnosed low-grade fibroblastic sarcoma [83]. Therefore, while true malignant transformation of desmoid-type fibromatosis may rarely occur, distinction from a pre-existing fibroblastic sarcoma remains an important diagnostic consideration, particularly in cases showing aggressive imaging features or clinicopathologic discordance, as illustrated in the present case. In this context, absence of nuclear β-catenin expression, *CTNNB1* alteration, and predisposing factors argues against fibromatosis. Instead, sarcoma should be considered, particularly when imaging demonstrates discordant aggressive features such as deep location, heterogeneity, or necrosis.

This case highlights the potential for diagnostic pitfalls related to tumor heterogeneity and limited biopsy representativeness, underscoring the importance of adequate tissue sampling and close multidisciplinary imaging and histologic assessment (Figure 11) [70,72]. In cases showing discordance between relatively bland histology and aggressive imaging findings, repeat biopsy, larger-volume sampling, or excisional biopsy should be considered. Multidisciplinary review integrating imaging, pathology, and clinical progression may substantially reduce the risk of delayed diagnosis.

Overall, these cases illustrate that apparent “malignant transformation” may occasionally reflect initial diagnostic underestimation rather than true biologic progression. Awareness of intratumoral heterogeneity, strategic biopsy targeting, and careful radiologic–pathologic correlation are therefore essential to avoid delayed diagnosis and inappropriate management in soft tissue tumors. More broadly, in heterogeneous soft tissue tumors, pre-procedure imaging can improve biopsy accuracy by identifying and targeting the most biologically aggressive regions, including enhancing, diffusion-restricted, or FDG-avid components, thereby reducing the risk of undersampling and diagnostic underestimation [84].

## 7. Patients at Risk for Malignant Transformation

Recognizing patients at high risk for malignant transformation and diagnostic pitfalls prone to underdiagnosis due to intratumoral heterogeneity is crucial for timely intervention (Table 1).

## 8. Conclusions

True malignant transformation, dedifferentiation, and secondary malignancy in soft tissue lesions represent a significant diagnostic challenge due to their subtle clinical progression and overlapping imaging and histopathologic features. As demonstrated through various tumorous and non-tumorous conditions—ranging from NF1-associated MPNSTs to chronic inflammation-induced malignancies—relying on a single diagnostic modality can lead to critical underdiagnosis, often exacerbated by tumor heterogeneity and sampling limitations. Therefore, a high index of clinical suspicion, particularly when faced with rapid growth, new-onset pain, or evolving imaging characteristics, is paramount. Ultimately, longitudinal radiologic surveillance with careful clinic radiologic–pathologic correlation may improve diagnostic accuracy and facilitate timely management. Referral to orthopedic tumor specialists or dedicated sarcoma centers, with multidisciplinary tumor board discussion involving radiology, pathology, orthopedic oncology, and oncology specialists, may further improve diagnostic accuracy and optimize patient management in these challenging cases.

Finally, we acknowledge the conceptual limitations of grouping diverse musculoskeletal conditions under the umbrella framework of “malignant progression.” Biologically, true malignant transformation, dedifferentiation, and secondary malignancies arising in chronically inflamed tissue represent fundamentally distinct processes with different underlying mechanisms. Although synthesizing these entities within a single framework provides educational value and may help radiologists structure diagnostic approaches, this approach inevitably simplifies biologically heterogeneous entities. Therefore, these lesions should be interpreted with careful consideration of their distinct pathophysiologic backgrounds and clinical contexts.

## Figures and Tables

**Table 1:** Risk factors for malignant transformation and diagnostic pitfalls in soft tissue lesions.

Category	Risk Factors	Key Warning Signs	Recommended Approach
Genetic predisposition	Neurofibromatosis type 1	Rapid growth, new pain, neurologic deficit; imaging: loss of target sign, internal heterogeneity, restricted diffusion on DWI	Close imaging surveillance, early biopsy, consider advanced imaging (DWI)
Chronic inflammation	Burn scars, chronic wounds, chronic empyema (TB pleurisy, therapeutic pneumothorax); long latency (20–40 years)	Ulceration, rapid growth, new solid component, invasion	Careful evaluation of enhancement pattern, assess invasion, low threshold for biopsy
Prior radiation exposure	Previous radiotherapy with long latency period (>5–10 years)	New enlarging enhancing soft tissue mass within irradiated field; heterogeneity, necrosis, invasion	Careful longitudinal comparison with prior imaging; low threshold for biopsy of new aggressive lesions within irradiated tissue
Benign or low-grade tumors with malignant potential	DFSP, SFT	Rapid interval growth, new-onset pain, increasing heterogeneity, fascia/muscle invasion	Longitudinal imaging comparison, re-biopsy if interval change
Diagnostic pitfalls: Intratumoral heterogeneity and undersampling	ALT/WDL: Large size (>10 cm), lower extremity or retroperitoneum, deep intramuscular, recurrence	Imaging: thick septa, septal/nodular enhancement, interval growth; pathology: increased fibrous septa with atypical stromal cells	Evaluate non-lipomatous components, *MDM2/CDK4* FISH if suspicious features
	High grade fibroblastic sarcoma: Deep extremity location, absence of fibromatosis predisposing factors (pregnancy, surgery, trauma, FAP)	Imaging: T2 signal heterogeneity, necrosis, marked interval enlargement; pathology: elevated Ki-67 index, absent nuclear β-catenin	Repeat biopsy or excisional biopsy when imaging findings are discordant with limited biopsy; β-catenin IHC, CTNNB1 mutation testing if β-catenin negative

## Data Availability

The data presented in this study are available on request from the corresponding author due to institutional and IRB-related restrictions.
